# ILC2 regulates hyperoxia-induced lung injury via an enhanced Th17 cell response in the BPD mouse model

**DOI:** 10.1186/s12890-023-02474-9

**Published:** 2023-05-30

**Authors:** Yue Zhu, Lanlan Mi, Hongyan Lu, Huimin Ju, Xiaobo Hao, Suqing Xu

**Affiliations:** 1 Department of Pediatrics, The Affiliated Hospital of Jiangsu University, No.438 Jiefang Road, Zhenjiang, Jiangsu 212001 China; 2 Department of Neonatology, Shanghai Children’s Medical Center, No.1678 Dongfang Road, Pudong New Area, Shanghai, 200127 China

**Keywords:** Bronchopulmonary dysplasia, Lung injury, Th17 cell response, Type 2 innate lymphoid cells

## Abstract

**Backgroud:**

Recent research has focused on the role of immune cells and immune responses in the pathogenesis of bronchopulmonary dysplasia (BPD), but the exact mechanisms have not yet been elucidated. Previously, the key roles of type 2 innate lymphoid cells (ILC2) in the lung immune network of BPD were explored. Here, we investigated the role Th17 cell response in hyperoxia-induced lung injury of BPD, as well as the relationship between ILC2 and Th17 cell response.

**Methods:**

A hyperoxia-induced BPD mouse model was constructed and the pathologic changes of lung tissues were evaluated by Hematoxylin-Eosin staining. Flow cytometry analysis was conducted to determine the levels of Th17 cell, ILC2 and IL-6^+^ILC2. The expression levels of IL-6, IL-17 A, IL-17 F, and IL-22 in the blood serum and lung tissues of BPD mice were measured by ELISA. To further confirm the relationship between ILC2 and Th17 cell differentiation, ILC2 depletion was performed in BPD mice. Furthermore, we used immunomagnetic beads to enrich ILC2 and then flow-sorted mouse lung CD45^+^Lin^-^CD90.2^+^Sca-1^+^ILC2. The sorted ILC2s were injected into BPD mice via tail vein. Following ILC2 adoptive transfusion, the changes of Th17 cell response and lung injury were detected in BPD mice.

**Results:**

The expression levels of Th17 cells and Th17 cell-related cytokines, including IL-17 A, IL-17 F, and IL-22, were significantly increased in BPD mice. Concurrently, there was a significant increase in the amount of ILC2 and IL-6^+^ILC2 during hyperoxia-induced lung injury, which was consistent with the trend for Th17 cell response. Compared to the control BPD group, ILC2 depletion was found to partially abolish the Th17 cell response and had protective effects against lung injury after hyperoxia. Furthermore, the adoptive transfer of ILC2 enhanced the Th17 cell response and aggravated lung injury in BPD mice.

**Conclusions:**

This study found that ILC2 regulates hyperoxia-induced lung injury by targeting the Th17 cell response in BPD, which shows a novel strategy for BPD immunotherapy.

**Supplementary Information:**

The online version contains supplementary material available at 10.1186/s12890-023-02474-9.

## Introduction

Bronchopulmonary dysplasia (BPD) is a serious respiratory disorder that has a negative impact on both the survival and prognosis of preterm infants [1, 2]. In order to improve the survival rate and long-term survival quality of preterm infants, in-depth research to explore the pathophysiology of BPD and develop preventative treatments is required.

The characteristic pathobiology of BPD are alveolar developmental arrest and lung tissue dysplasia. Northway et al. [3] hypothesized that there are four major elements in BPD pathogenesis: lung immaturity, respiratory failure, oxygen supplementation, and positive pressure mechanical ventilation. One of the most critical variables in the development of BPD is exposure to high concentrations of oxygen, as the severity of lung injury depends on the duration of oxygen exposure and the degree of positive pressure during mechanical ventilation [4, 5]. Although the processes and functions of distinct immune cells require further elucidation, the change ratio of immune cells also has a significant role in the pathogenesis of BPD [6, 7].

T helper 17 (Th17) cells are one of the CD4^+^T cell subsets. Th17 cell produces interleukin (IL)-17 (IL-17 A, IL-17 F), IL-22, and IL-26 in response to TGF-β and IL-6 [8, 9]. Recent research has found that placental cord blood samples from very preterm neonates with BPD contain more TEM, precursor Th17, mature Th17, and IL-17^+^Treg lymphocytes [10]. This was accompanied by an increase in the IL-6 concentration in neonatal tracheal aspirates, which was linked to the progression of BPD [11, 12]. Our previous studies found that that lung inflammation was significantly reduced in BPD mice after the administration of anti-IL-17 neutralizing antibody [13]. Therefore, we concluded that IL-17 and the Th17 cell response play an important role in BPD development. However, the specific mechanism is still unclear and needs to be further explored.

Innate lymphoid cells (ILCs) are innate immune system effector cells and they are divided into three primary subpopulations: ILC1, ILC2, and ILC3 [14]. In response to stimulation by IL-33, IL-25, and thymic stromal lymphopoietin (TSLP), type 2 innate lymphoid cells (ILC2) release Th2 cytokines, which are implicated in the development of type 2 immune responses in the lung [15, 16]. In adult mice, most ILC2 was found to be generated during the neonatal period, with only a minor amount gradually being replaced by freshly produced ILC2 [17], indicating that the neonatal period is crucial for ILC2 formation. Our previous study found that ILC2 is activated in large amounts and produces excess Th2 type cytokines under the stimulation of hyperoxia, inflammation, and mechanical ventilation in preterm infants. The activated ILC2 activates the lung immune network, leading to the interruption of inflammatory cascade response and alveolarization process, which ultimately promotes the development of BPD [18]. In this study, we found that IL-6 is highly expressed in ILC2 of BPD mice, and Th17 cell differentiation was directly correlated to increased IL-6 expression in ILC2. Qiu et al. [19] found that there was a significant expression congruence between ILC2, IL-6, and Th17 cell development and the protective impact of IL-33 deficiency in colitis. Accordingly, we hypothesized that hyperactive Th17 cell responses in BPD are related to ILC2 activation, and that ILC2 is involved in the BPD disease process through the modulation of Th17 cell responses.

Overall, this study found a hyperactivity Th17 cell response in hyperoxia-induced BPD mice, which was attributed to ILC2 activation. Furthermore, ILC2 depletion and adoptive transfusion were found to demonstrate the role of ILC2 in hyperoxia-induced lung injury of BPD through the modulation of Th17 cell responses. This study sheds light on immunotherapy strategies for BPD and provides insight into the immune regulatory mechanisms of hyperoxia-induced lung injury in BPD.

## Materials and methods

### Mice and BPD model

All the animal experimental protocols were reviewed and approved by the Animal Ethics Committee of Jiangsu University (protocol No. UJS-IACUC-AP-2,020,030,304). C57BL/6 mice were provided by the Animal Center of Jiangsu University (Zhenjiang, China), and the BPD mice model was established according to previous studies [20, 21]. The lung development of neonatal mice is equivalent to that of human lungs gestational age of 28 weeks, which can well simulate the lung development of human preterm infants. Therefore, we selected full-term neonatal mice to construct a BPD model. Newborn mice were randomly numbered and assigned to equal-sized litters in odd or even numbers, and and placed into either a normoxia or hyperoxia environment within two hours of birth. Mouse pups were exposed to the 85% O2 oxygen concentration starting on the day of birth, continuously up to and including day 14. For oxygen-exposure protocols, nursing dams were rotated every 12 h. This addresses the oxygen toxicity issues in adult mice, which are highly susceptible to prolonged periods of hyperoxia. Nursing dams received food ad libitum. Mice were maintained in a 12 h:12 h dark/light cycle. No mice died during the experiment in each group. All animal experiments were performed in accordance with Guide and Care and Use of Laboratory Animals from National Institutes of Health (NIH) and ARRIVE. Throughout the entire experimental process, we identified pain and suffering, alleviated or prevented the development of pain and/or suffering, and adopted measures such as improving the living environment, diet, or medication intervention if necessary.

### Flow cytometry analysis

Lung tissues were sectioned into small pieces and 1 mg/mL collagenase A (Roche, Basel, CH) was added and digested at 37 °C for 30 min. The digestion solution was filtered through a 70 μm cell strainer (BD, Franklin Lake, NJ, USA) to obtain a single-cell suspension. To identify ILC2, the following antibodies were used: BV510 anti-CD45 (cat.no.103,138; BioLegend), FITC anti-Lineage (cat.no.133,302; BioLegend), PE anti-CD90.2(cat.no.105,308; BioLegend), APC anti-Sca-1(cat.no.E-AB-F1191UE; Elabscience). The Lineage mixture includes CD3, Ly-6G/Ly-6CT, CD11b, CD45R/B220 and TER-119/Erythroid cells. To identify the Th17 cells, we used BV510 anti-CD45, PE anti-CD4(cat.no.MA5-17451, invitrogen) and FITC anti-IL-17 A (cat.no.11-7177-81, invitrogen). Intracellular staining was also performed to assess cytokine production using IL-6 (eFluor^TM^450, cat.no.48-7061-82, invitrogen) and IL-17 A after 4 h of in vitro incubation with 50 µg/mL PMA (Sigma-Aldrich, St. Louis, MO, USA), Acquisition was performed on a BD FACS Canto (BD, Franklin Lake, NJ, USA) using the BD FACSDiva software v8.0.1. Data were analyzed with FlowJo software (TreeStar, BD Biosciences, Franklin Lake, NJ, USA) version 10.

### ILC2 depletion

For ILC2 depletion, neonatal mice were administered anti-CD90.2 antibody (cat.no.E-AB-F10940; BioLegend) during hyperoxia exposure[18, 22]. Mice in the hyperoxia group were injected intraperitoneally with anti-CD90.2 antibody at 7 and 10 d postnatally (50 µg/day) and control animals were injected with the control IgG. Mice in the different groups were sacrificed at day 14 to evaluate the depletion of ILC2 in BPD.

### ILC2 sorting and adoptive transfusion

The lung tissues of neonatal mice were collected under sterile conditions, and ILC2 enriched cell suspensions were prepared according to the instructions of mouse ILC2 enrichment Kit (STEMCELL Technologies Inc., CA). Subsequently, mouse lung ILC2(CD45^+^Lin^-^CD90.2^+^Sca-1^+^) were sorted using a BD FACS Aria II flow cytometer (BD, Franklin Lake, NJ, USA). The sorted ILC2s were incubated with 5 µg/mL Cy7 Dic18 fluorescent cyanine dye (MCE, NJ, USA) and then injected intravenously into neonatal mice at day 7 (3 × 10^5^ cells/mouse). ILC2 adoptive transfusion were obtained with the previously-described method and optimize the operation [23, 24]. The whole-body imaging was performed using a Spectrum CT machine (PerkinElmer, Waltham, MA, USA) at 0, 6, and 30 h after ILC2 adoptive transfusion in the hyperoxia group. BPD mice were sacrificed to harvest the heart, lung, liver, spleen, kidney, and stomach, and ex vivo imaging of the organs was performed. Mice in the different groups were sacrificed at day 14 to evaluate the ILC2 adoptive transfusion in BPD.

### Histological analysis

Paraformalde-fixed lung tissues were dehydrated with alcohol and xylene to prepare 3-µm sections for subsequent experiments. The sections were used for hematoxylin and eosin (H&E) staining for morphometric analysis. Radial alveolar counts (RAC) and lung injury scores were obtained using a previously-described method [25–27]. For lung injury scores, each image is evaluated by three inspectors who do not know the identity of the slide, and the average value is taken as the lung injury score for that image. We analyzed 6 images per group. The components of the lung injury score includes alveolar capillary congestion, hemorrhage, infiltration or aggregation of inflammatory cells in the airspace or interstitium, as well as the thickness of the alveolar wall/hyaline membrane formation. Each characteristic was scored from 0 to 3, including 0 = absence, 1 = mild, 2 = moderate and 3 = prominent.

### Periodic acid-Schiff (PAS) staining

The lung tissues were fixed with 4% paraformaldehyde at 4 ˚C overnight, washed with PBS and processed into paraffin blocks. Tissues sections were stained with periodic acid-Schiff (PAS) reagent (Beijing Solarbio Science & Technology Co, Beijing, China) for analyzing the content of glycogen. Positive glycogen staining was visualized in red or purple color. The sections were observed under a light microscope and the images were analyzed by Image-Pro Plus III (Media Cybernetics, Inc., Rockville, MD, USA) to obtain the mean gray value.

### Wright-Giemsa staining

The sorted ILC2 cell suspension was evenly smeared on a glass slide and stained with Wright-Giemsa stain solution (Solarbio, Beijing, China). After 1–2 min, we added an equal amount of 0.01 M disodium hydrogen phosphate solution to the smear, mixed it with the Wright-Giemsa stain solution thoroughly, stained it for 3–5 min and then flushed it with water. The morphology of ILC2was observed under light microscope.

### Reverse transcription-quantitative (RT-q) PCR

Total RNA was extracted from ILC2 using the TRIzol reagent (Invitrogen, Carlsbad, CA, USA), and the total RNA concentration was determined in accordance with the instructions of the reverse transcription kit (Thermo Fisher Scientific, Waltham, MA, USA), and the first strand of cDNA was synthesized. Quantitative analysis was performed using a Roche Light Cycler Sequence Detection System (Roche, Basel, CH). The amplification program was 95 °C for 30 s, 40 cycles of 95 °C for 30 s, and 60 °C for 34 s. The IL-6 primer sequence was as follows: forward: 5′- cacaagtccggagaggagac − 3′, reverse: 5′- ttgccattgcacaactcttt − 3′. β-actin was used as the internal control. The RT-qPCR was conducted with three independent replicates and the relative gene expression level was analyzed using the comparative 2^−ΔΔCT^ method [28].

### ELISA

IL-17 A, IL-17 F, and IL-22 levels in the blood serum and lung tissue samples were assayed using ELISA kit (Multi Sciences (Lianke) Biotech Co.,Ltd, Hangzhou, China), as well as IL-6 levels were analyzed using an ELISA kit (eBioscience, CA, USA) for Cytokine quantification. ELISA was performed in accordance with the manufacturer’s protocol.

### Statistical analysis

All experiments were repeated 6 times (n = 6). Results are presented as the mean ± standard deviation (SD)and in accordance with the normal distribution. All data were analyzed using SPSS 22.0 (IBM, SPSS, Chicago, IL, USA). Comparisons between two groups were performed using the independent samples t-test, while comparisons among multiple groups were performed using one-way analysis of variance (ANOVA) with Tukey’s multiple comparison post hoc test. *P* < 0.05 was considered statistically significant, 95% Confidence Interval.

## Results

### Lung injury and Th17 cell response in BPD mice

**Fig. 1:**
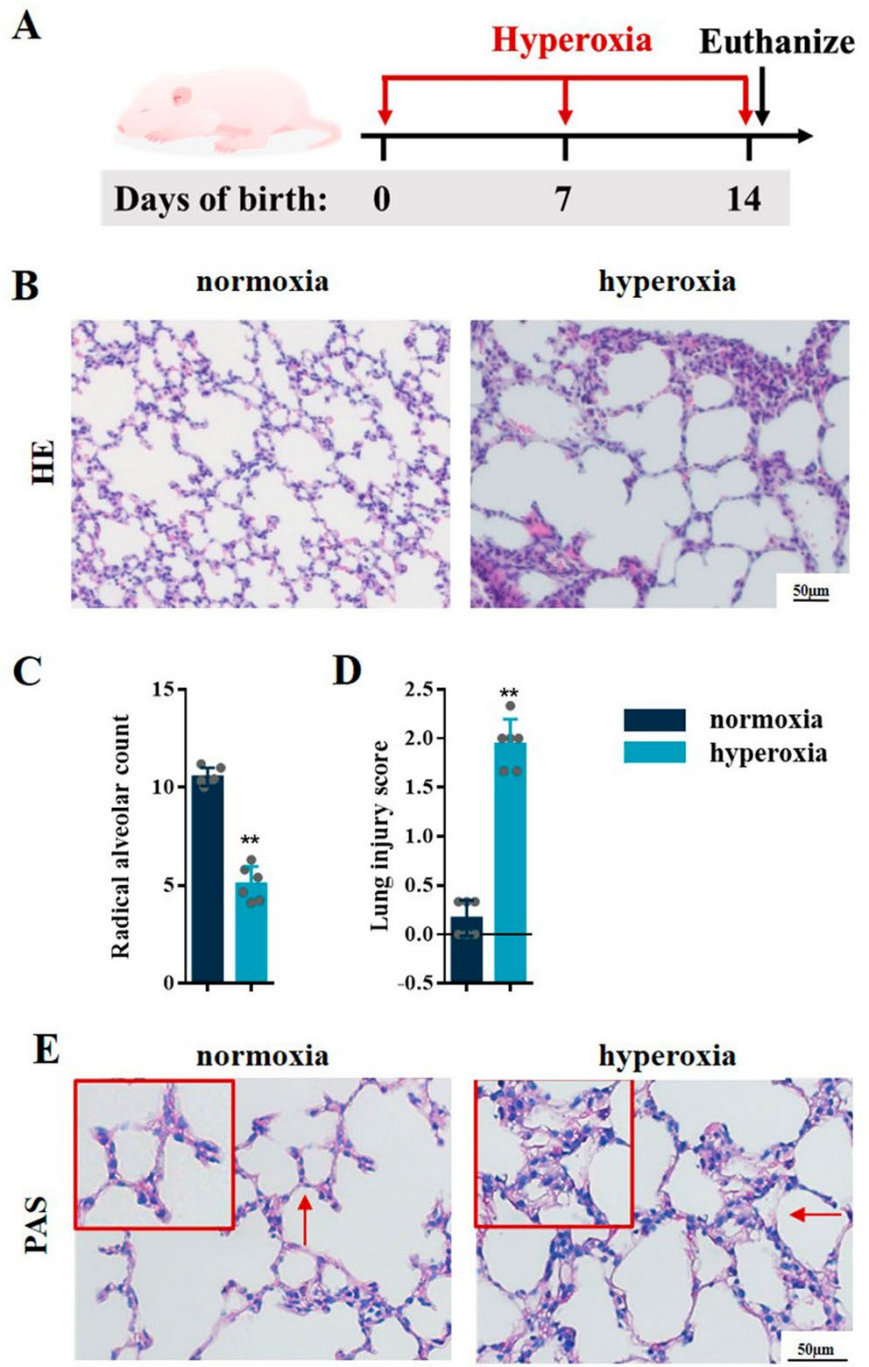
Hyperoxia induced lung injury in BPD mice. **A** C57BL/6 newborn mice were treated with normoxia or hyperoxia exposure from birth. Neonatal mice from the different groups were sacrificed at day 14. **B** Representative H&E staining of the lungs from the neonatal mice in the normoxia and hyperoxia groups. **C, D** lung injury was assessed by RAC and lung injure score. **E** Representative PAS staining of the lungs from the neonatal mice in the normoxia and hyperoxia groups. Data presented are the mean ± SD (n = 6); ** *P* < 0.05 vs. normoxia group

As shown in Fig. 1A, a hyperoxia-induced BPD mouse model was constructed and the pathologic changes of lung tissues were evaluated by H&E staining. In hyperoxia group, the alveolar structure was disordered, the number of alveoli was reduced, the structure was simplified, the alveolar wall was gradually thickened, and the RAC was reduced (Fig. 1B C); this was similar to the pathological changes in human BPD [2]. The lung injury scores were also increased in BPD mice (Fig. 1D). The epithelial content of glycogen reflects the development and maturity of the lung. Studies have found that glycogen is abundant in immature alveolar type II cells [29, 30]. As the lung gets matured, the intracellular glycogen is gradually consumed and transformed into pulmonary surfactant phospholipids or lamellar bodies to maintain lung function after birth. Glycogen accumulates when the alveolar type II cell differentiation is impeded, but decreases as the lung develops. Therefore, the content of glycogen can reflect in the development of lung tissues as a reference indicator [31]. As shown in PAS staining, the glycogen appeared as red or purple color. Persistent hyperoxia exposure in neonatal mice resulted in marked changes in the content of glycogen. Compared with the room air littermates, the content of glycogen increased in hyperoxia-exposed mice (Fig. 1E).

**Fig. 2:**
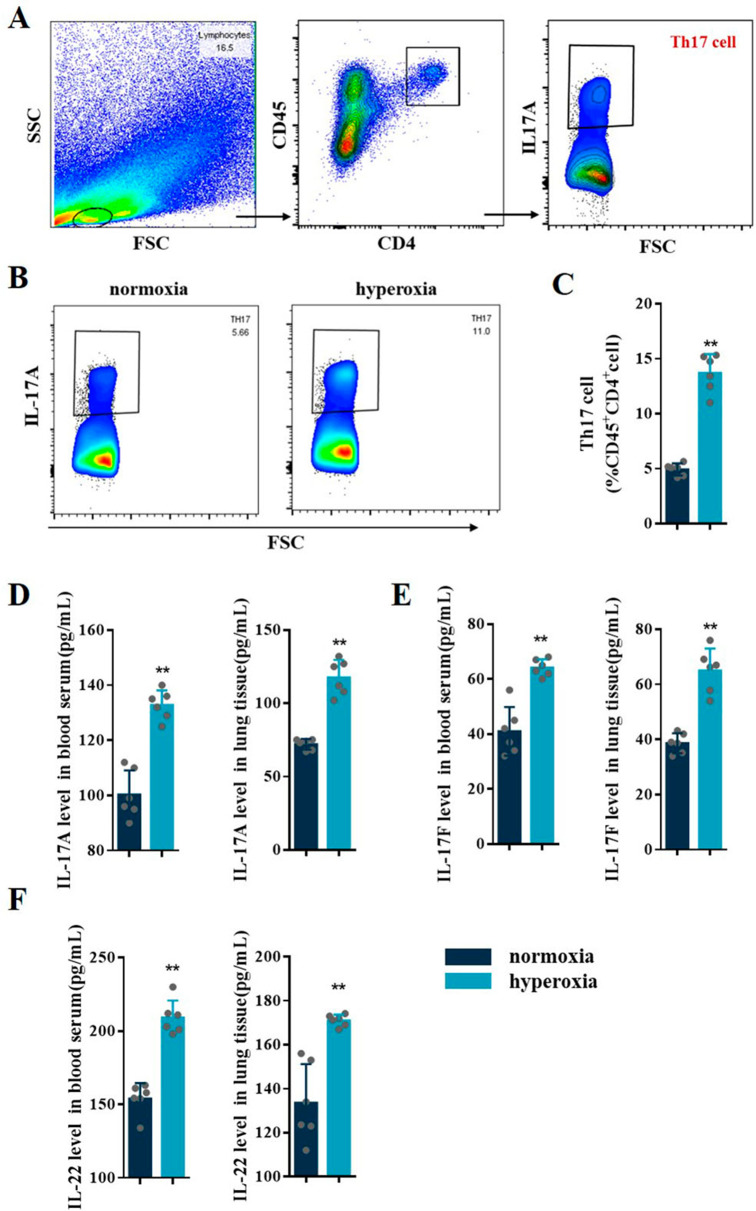
Hyperoxia induced Th17 cell response in BPD mice. **A** Representative FACS analysis showing the gating strategy used to identify CD45^+^CD4^+^IL-17 A^+^Th17 in lung of BPD mice. **B** Representative results of Th17 cells detected by flow cytometry in lung tissues from the normoxia and hyperoxia groups. **C** Percentage of Th17 cells detected by flow cytometry in lung tissues from the normoxia and hyperoxia groups. **D–F** IL-17 A, IL-17 F, and IL-22 levels in the blood serum and lung tissues were assessed in the normoxia and hyperoxia groups. Data presented are the mean ± SD (n = 6); ** *P* < 0.05 vs. normoxia group

Further, the level of Th17 cells (CD45^+^CD4^+^IL-17 A^+^) was significantly increased in lung tissue of BPD mice (*P* < 0.05) (Fig. 2A C). ELISA results showed that the expression levels of Th17 cell-related cytokines, including IL-17 A, IL-17 F, and IL-22, were significantly increased in the blood serum and lung tissues of BPD mice (Fig. 2D F), suggesting that the Th17 cell response is hyperactive in BPD.

### ILC2 and IL-6^+^ ILC2 in lung tissues of BPD mice

**Fig. 3:**
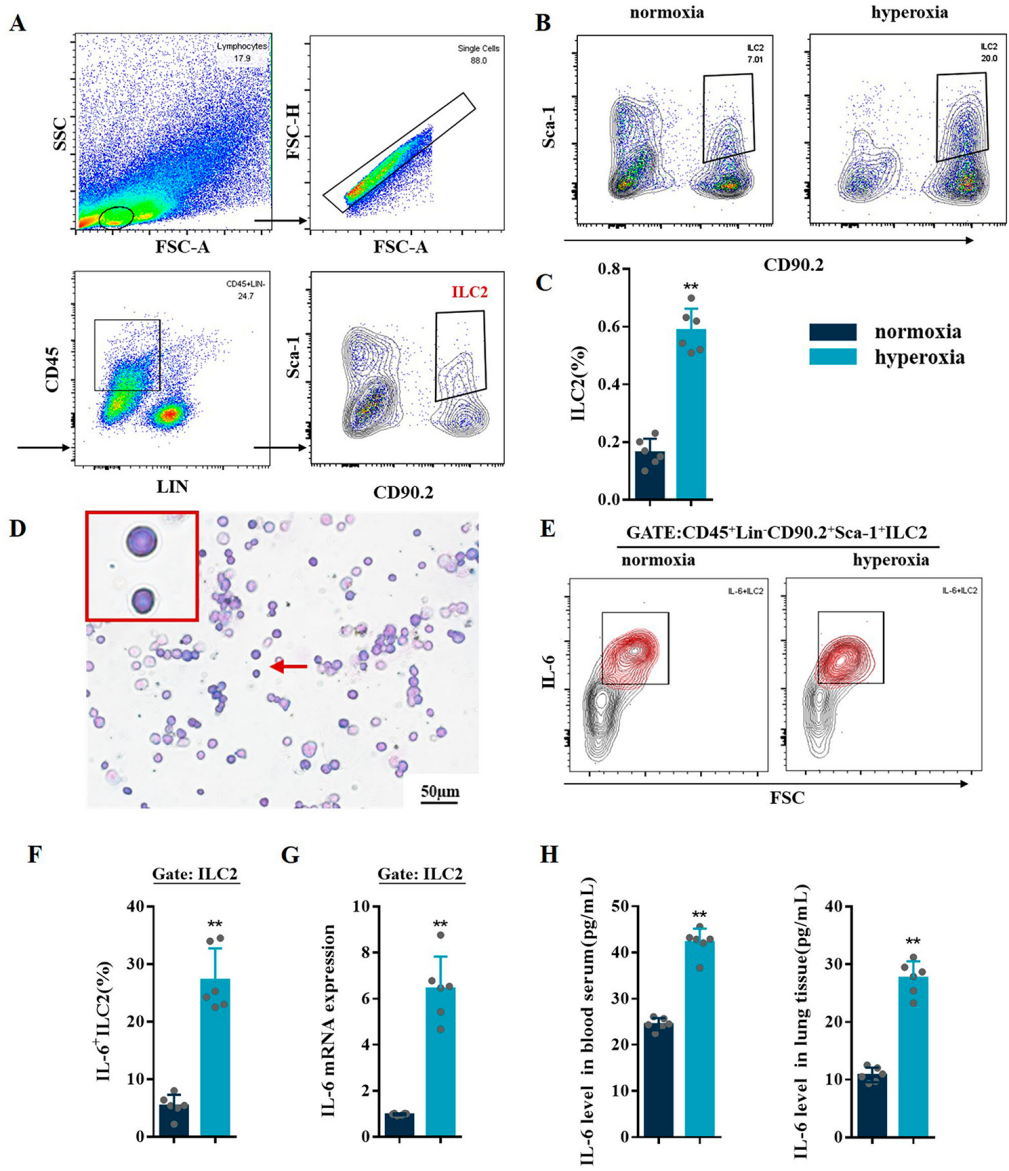
Hyperoxia induced ILC2 and IL-6^+^ILC2 in BPD mice. **A** Representative FACS analysis showing the gating strategy used to identify CD45^+^Lin^-^CD90.2^+^Sca-1^+^ILC2 in lung of BPD mice. **B, C** Representative results and percentage of ILC2 detected by flow cytometry in lung tissues from the normoxia and hyperoxia groups. **D** Representative Wright-Giemsa staining of sorted ILC2. **E, F** Representative results and percentage of IL-6^+^ILC2 (gate: CD45^+^Lin^-^CD90.2^+^Sca-1^+^) detected by flow cytometry in normoxia and hyperoxia groups. **G** RT-qPCR analysis of IL-6 mRNA levels in ILC2 of normoxia and hyperoxia groups. **H** ELISA analysis of IL-6 expression in the blood serum and lung tissues from the normoxia and hyperoxia groups. Data represented as mean ± SD (n = 6); ** *P* < 0.05 vs. normoxia group

Flow cytometry was used to detect the ILC2 (CD45^+^Lin^−^CD90.2^+^Sca-1^+^) expression levels in the lung tissues. There was a significant increase in the amount of ILC2 during hyperoxia-induced lung injury (Fig. 3A C), which was consistent with the trend for Th17 cell response. We used immunomagnetic beads to enrich ILC2 and then flow-sorted mouse lung CD45^+^Lin^−^CD90.2^+^Sca-1^+^ILC2. We used FMO controls in combination with isotype controls to check for non-specific binding of antibodies in the FACS (Supplementary Fig. 1). The selected cells were stained with Wright-Giemsa staining. We found that the cells were oval or round, with small cytoplasmic areas and large nucleus. The nuclear chromatin was dense and consistent with the characteristics of lymphocytes (Fig. 3D). Flow cytometry also verified that IL-6 level was significantly increased in sorted ILC2 of BPD mice (Fig. 3E F), which was further confirmed by RT-qPCR (Fig. 3G). ELISA results showed that IL-6 expression was significantly upregulated in the blood serum and lung tissues during BPD (Fig. 3H).

### ILC2 depletion suppresses Th17 cell responses and lung injury in BPD mice

**Fig. 4:**
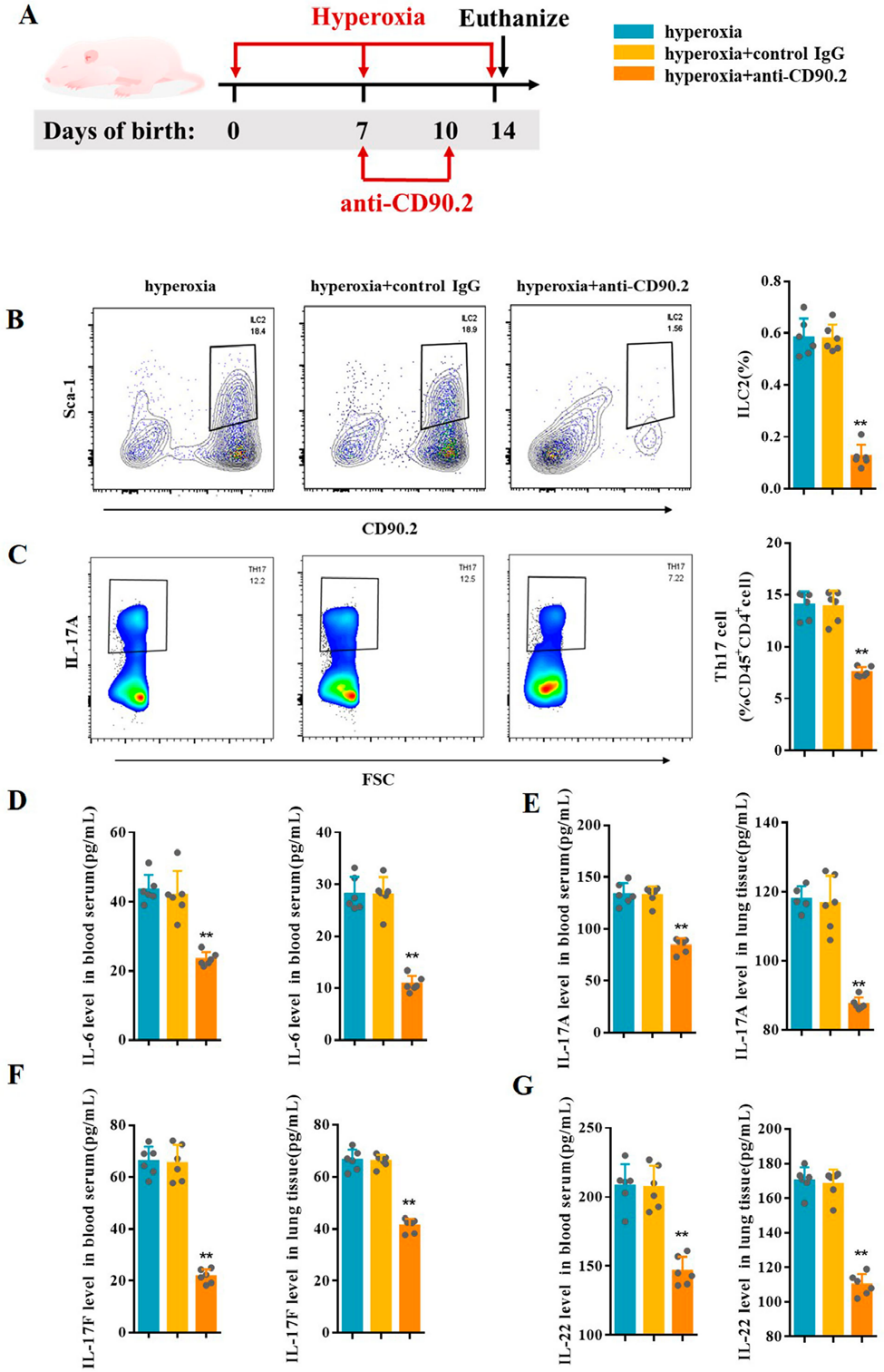
ILC2 depletion inhibited Th17 cell response. **A** Newborn mice were treated with anti-CD90.2 antibody or control IgG twice during hyperoxia exposure. Neonatal mice in different groups were sacrificed at day 14. **B** Representative results and the percentage of ILC2 detected by flow cytometry in the lungs of the hyperoxia, hyperoxia + control IgG, and hyperoxia + anti-CD90.2 groups. **C** Representative results and percentage of Th17 cells detected by flow cytometry in lung samples of the hyperoxia, hyperoxia + control IgG, and hyperoxia + anti-CD90.2 groups. **D–G** IL-6, IL-17 A, IL-17 F, and IL-22 levels in the blood serum and lung tissues were assessed in the hyperoxia, hyperoxia + control IgG, and hyperoxia + anti-CD90.2 groups. Data presented are the mean ± SD (n = 6); ** *P* < 0.05 vs. hyperoxia group

To assess the potential role of ILC2 on Th17 cell response, anti-CD90.2 antibody were used for ILC2 depletion [18] (Fig. 4A). Compared to mice in hyperoxia group and control IgG group, the results of flow cytometry analysis confirmed ILC2 depletion in BPD mice treated with anti-CD90.2 antibody (Fig. 4B). In addition, CD45^+^CD4^+^IL-17 A^+^Th17 cells decreased significantly following ILC2 depletion (Fig. 4C). ELISA results showed that IL-6, IL-17 A, IL-17 F, and IL-22 were significantly reduced in the blood serum and lung tissues of mice after ILC2 depletion (Fig. 4D and G).

**Fig. 5:**
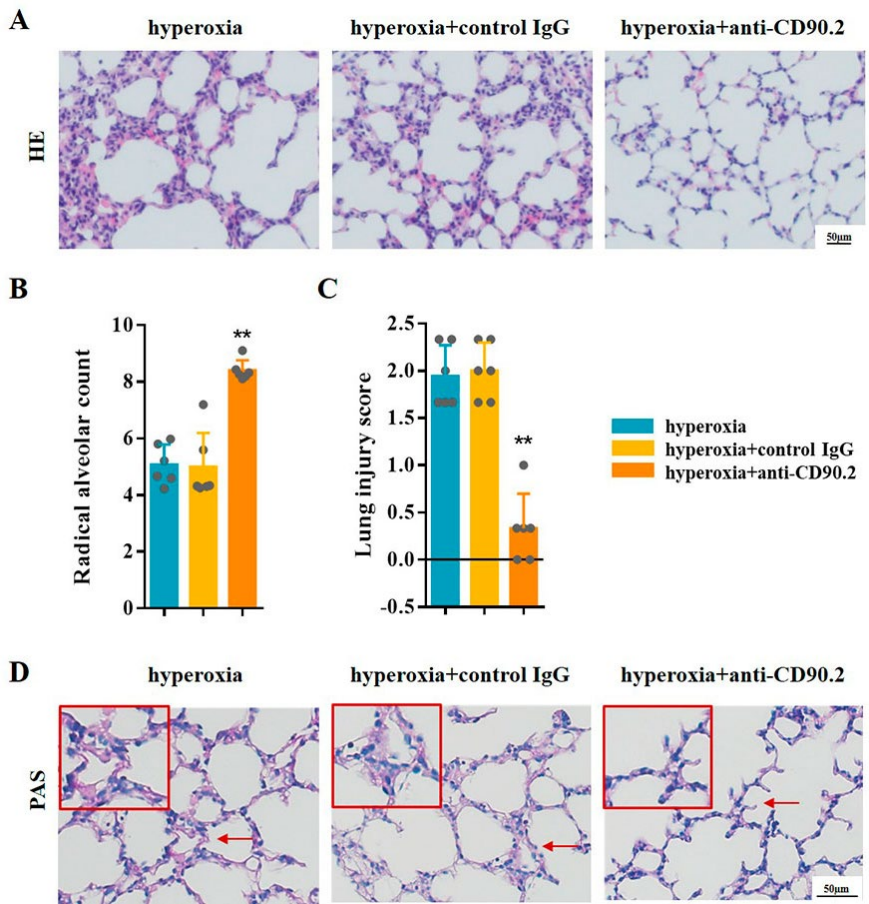
ILC2 depletion inhibited hyperoxia-induced lung injury in BPD mice. **A** Representative H&E staining of the lung samples from the neonatal mice in the hyperoxia, hyperoxia + control IgG, and hyperoxia + anti-CD90.2 groups. **B, C** lung injury was assessed by RAC and lung injure score. **D** Representative PAS staining of the lungs from the neonatal mice in the hyperoxia, hyperoxia + control IgG, and hyperoxia + anti-CD90.2 groups. Data presented are the mean ± SD (n = 6); ** *P* < 0.05 vs. hyperoxia group

H&E staining showed that the lung tissue structure of BPD mice became regular, the size of alveolar cavity was more uniform, and the number of alveoli increased after treatment with anti-CD90.2 antibody (Fig. 5A). Following ILC2 depletion, RAC was increased and lung injury scores were decreased (Fig. 5B C). Glycogen of mice lung reduced after ILC2 depletion, while there was no difference between hyperoxia group and control IgG group (Fig. 5D). These results suggested that ILC2 depletion alleviates lung development arrest and suppresses hyperoxia-induced lung injury in BPD mice model effectively.

### ILC2 adoptive transfusion aggravates Th17 cell response and lung injury in neonatal mice

**Fig. 6:**
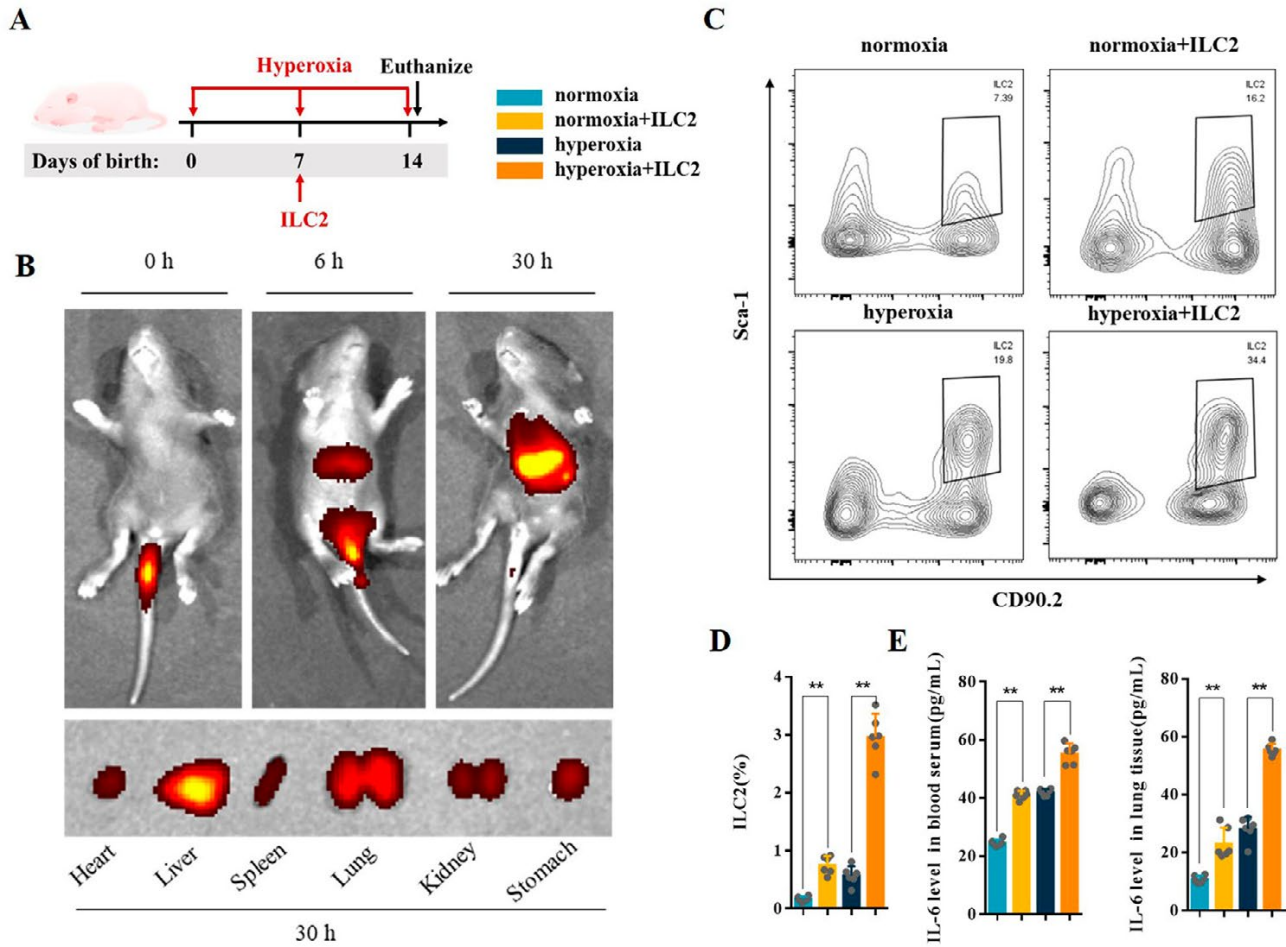
ILC2 adoptive transfusion in neonatal mice. **A** Neonatal mice were treated with an adoptive transfusion at day 7 during hyperoxia exposure. Neonatal mice in different groups were sacrificed at day 14. **B** In vivo imaging of Cy7-stained ILC2 in BPD mice; upper panel: whole-body imaging at 0, 6, and 30 h; lower panel: ex vivo imaging of the heart, lung, liver, spleen, kidney, and stomach at 30 h. **C, D** Representative results and percentage of ILC2 detected by flow cytometry in the lung tissues from the different groups. **E** IL-6 levels in the blood serum and lung tissues were assessed in different groups. Data presented are the mean ± SD (n = 6); ** *P* < 0.05

**Fig. 7:**
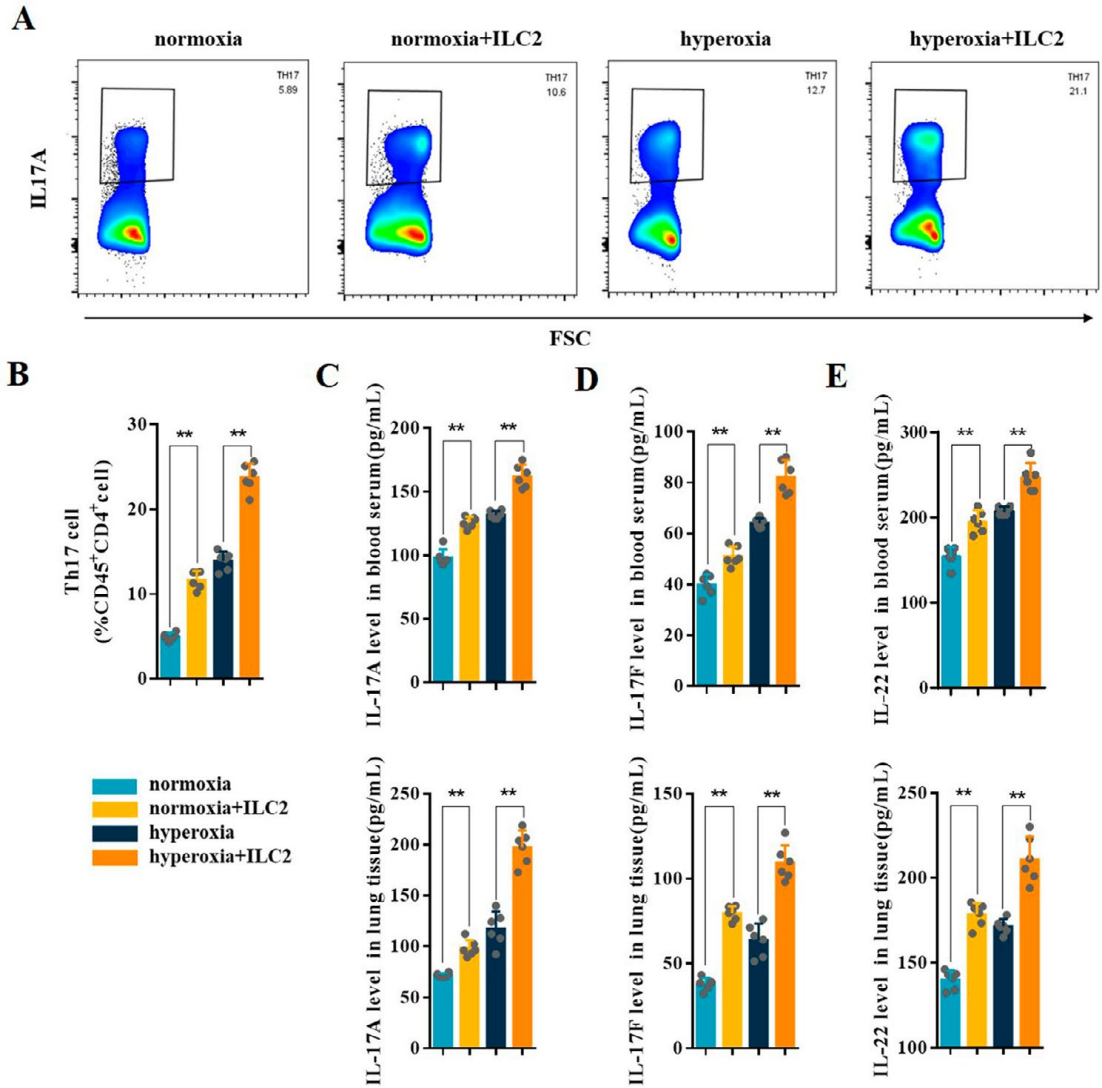
ILC2 adoptive transfusion enhanced the Th17 cell response in neonatal mice. **A, B** Representative results and the percentage of Th17 cells detected by flow cytometry in the lung tissues from the different groups. **C–E** IL-17 A, IL-17 F, and IL-22 levels in the blood serum and lung tissues were assessed in the different groups. Data presented are the mean ± SD (n = 6); ** *P* < 0.05

As shown in Fig. 6A, the sorted ILC2s were injected into normoxia or hyperoxia mice via tail vein at day 7. Cy7 Dic18-stained ILC2s were observed in the heart, liver, lung, kidney, and stomach at 30 h after injection during hyperoxia exposure and were mainly concentrated in the liver and lung (Fig. 6B). This suggests that hyperoxia can induce ILC2 recruitment in lung. Flow cytometry analysis of CD45^+^Lin^−^CD90.2^+^Sca-1^+^ILC2 confirmed that ILC2 transfusion was successful (Fig. 6C and D). The expression level of IL-6 was significantly increased in the blood serum and lung tissues of normoxia and hyperoxia mice after ILC2 transfusion (Fig. 6E). Following ILC2 adoptive transfusion, CD45^+^CD4^+^IL-17 A^+^Th17 cells increased significantly both in normoxia and hyperoxia mice (Fig. 7A and B). ELISA results showed that IL-17 A, IL-17 F, and IL-22 were significantly increased in the blood serum and lung tissues of normoxia and hyperoxia mice after ILC2 transfusion (Fig. 7C and E).

**Fig. 8:**
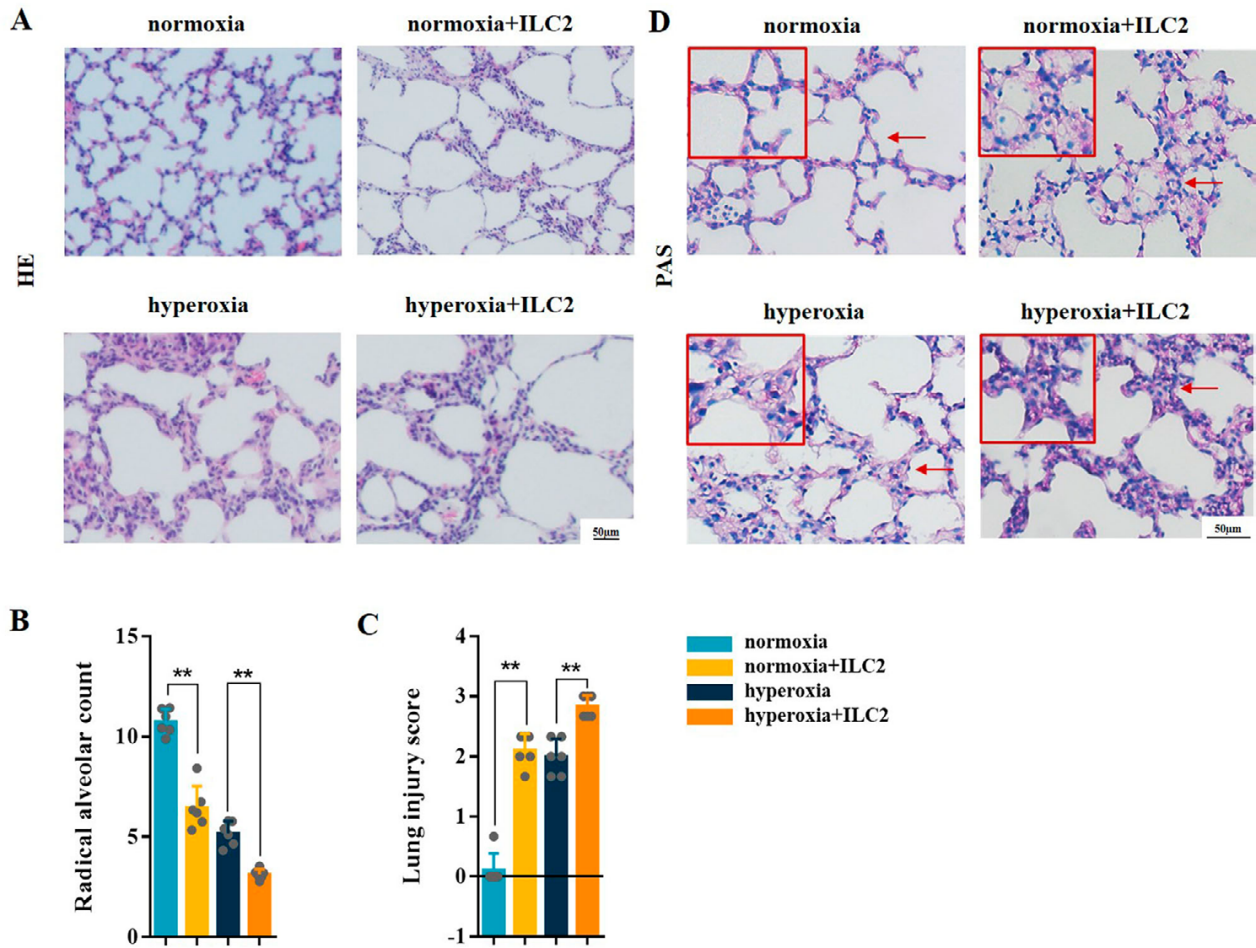
ILC2 adoptive transfusion enhanced lung injury in neonatal mice. **A** Representative H&E staining of the neonatal lung tissues from the mice in the different groups. **B, C** lung injury was assessed by RAC and lung injure score. **D** Representative PAS staining of the lungs from the neonatal mice in the different groups. Data presented are the mean ± SD (n = 6); ** *P* < 0.05

H&E staining showed that after ILC2 adoptive transfusion, the alveoli of the normoxia mice were further fused, lung structures had become more simplified and disorganized, which were similar to the pulmonary manifestations of BPD. After adoptive infusion of ILC2 into the hyperoxia group mice, the development of alveoli was further aggravated (Fig. 8A). Following ILC2 transfusion, RAC was decreased and lung injury scores were increased in both normoxia and hyperoxia mice (Fig. 8B C). PAS staining showed an increase in lung glycogen levels in both the normoxia and hyperoxia mice after ILC2 transfusion(Fig. 8D). These results suggested that ILC2 adoptive transfusion exacerbates lung development arrest and aggravates hyperoxia-induced lung injury in BPD mice model.

## Discussion

In this study, we found that the Th17 cell response was hyperactive and involved in the development of hyperoxia-induced lung injury of BPD. Furthermore, we have provided evidence that ILC2 has a role in Th17 cell differentiation by ILC2 depletion and adoptive transfusion, which helps to identify the importance of the ILC2-Th17 immune axis in hyperoxia-induced lung injury in BPD.

Apart from higher concentrations of leukotriene, endothelin-1[32], IL-6[33], and IL-17 A [34, 35], newborns destined for BPD also exhibit an increase in the number of circulating neutrophils and macrophages. During the alveolar phase (lung development is not mature), the increase in IL-17 A activates the IL-17 signaling pathway, causing inflammation, which may further affect lung development and lead to the occurrence of BPD [36]. Recent studies have found that IL-17 A level is significantly upregulated and the IL-17 signaling pathway is significantly activated in the external circulation of children with BPD [10, 34, 35]. In the BPD mouse model, the expression of Th17 cells was enhanced in lung, demonstrating strong pro-inflammatory properties and resistance to Treg mediated inhibition [13, 37, 38]. During the critical developmental window of premature infants, the Th17 biased pro-inflammatory response caused by intrauterine inflammation weakens the immune program, leading to “dysfunctional immune activation“[39]. This uncontrolled immune regulation ultimately leads to severe inflammatory processes and tissue damage, which in turn increases the incidence rate and mortality associated with BPD and chronic lung disease [40].

In this study, Th17 cells were found to be increased in the lung tissues of BPD mice. The expression of IL-17 A, IL-17 F, and IL-22 was also significantly increased in blood serum and lung tissues, indicating that Th17 cell response is hyperactive in BPD. This suggested that excessive activation of Th17 cell response disrupts normal lung development and participates in the development of BPD. In our previous study, we used anti-IL-17 neutralizing antibody to inhibit IL-17 expression in BPD mice and found that lung inflammation in BPD was reduced after IL-17 intervention (13). These results further demonstrated the destructive role of IL-17 and the hyperactive Th17 cell response in BPD.

However, as an upstream regulator of Th17 cell differentiation, the role of IL-6 in neonatal lung injury has been controversial. Huusko et al. [41] showed that the expression of IL6, IL6R, and IL6ST in the cord blood of preterm infants was not associated with the development of BPD. Other clinical studies have shown that elevated concentrations of IL-6, sIL-6R, and sgp130 in tracheal aspirates from infants are significantly associated with BPD [11, 42, 43]. The contradiction may be due to the fact that plasma does not necessarily reflect changes in intrinsic IL-6 expression in the lungs. Therefore, tracheal aspirates may be more suitable for exploring the trend in IL-6 level in BPD. Hirani et al. [44] further demonstrated that the IL-6 knockout protected neonatal mice from hyperoxia-induced lung injury and promoted the development of alveolar production. Similarly, the present study showed that IL-6 expression is significantly increased in the blood serum and lung tissues of BPD mice, which is consistent with hyperoxia-induced lung injury, and directed the immune response towards the Th17 phenotype. Our current findings and previous studies have emphasized the importance of strictly regulating IL-6 and the Th17 cell response during window period of lung development.

There is a paucity of research on the source of IL-6 in BPD. Previous studies have showed that macrophage-derived IL-6 trans-signaling exacerbates alveolar epithelial inflammation, injury and impairs alveolar differentiation, which ultimately leads to the development of BPD [44]. In this study, we found that ILC2 is also an important source of IL-6 in BPD. This result was verified by RT-qPCR and flow cytometry, because IL-6 was found to be significantly upregulated in ILC2 of hyperoxia-injured lung. Additionally, IL-6 expression was significantly reduced after ILC2 depletion, but was significant increased after ILC2 adoptive transfer to the lungs. In brief, these results suggested that ILC2-derived IL-6 plays an important role in BPD and directs an immune response to the Th17 cells.

**Fig. 9:**
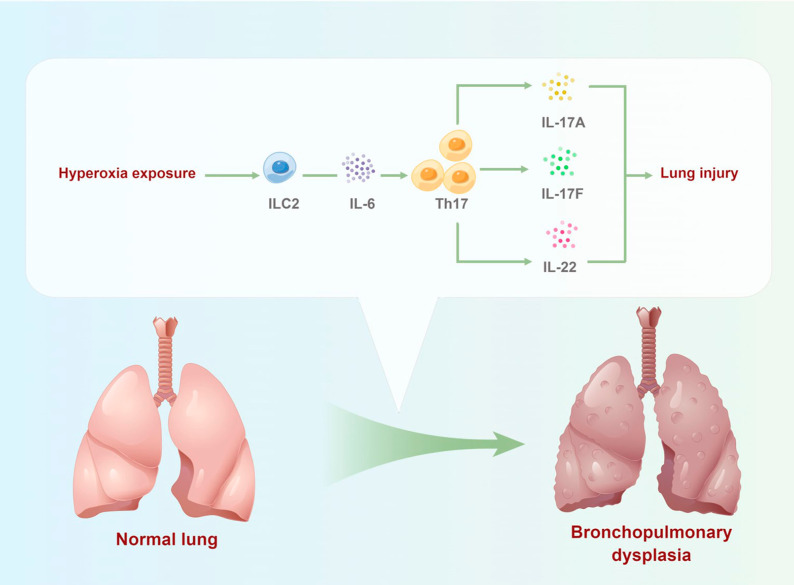
Schematic representation of the mechanism of ILC2 on regulating hyperoxia-induced lung injury by targeting Th17 cell response in BPD mice model

The lung microenvironment is critical for controlling the optimal immune response, as abnormal or excessive control can be detrimental to the organism [45]. ILC2, a major participant in lung immune microenvironment. Previous studies have shown that ILC2 plays an important role in the normal development of fetal lungs and BPD lung differentiation [18, 46]. However, the mechanism of activating immune network and expanding cascade response remains to be elucidated. In this study, we found that after ILC2 adoptive reinfusion, normoxia mice showed BPD-like lung injury. In addition, IL-6 is highly expressed in ILC2 of BPD mice, and Th17 cell differentiation was directly correlated to increased IL-6 expression in ILC2. It is worth noting that the Th17 cell response was significantly altered in both ILC2 depletion and adoptive transfusion experiments. These results indicated that hyperoxia induce ILC2 to secrete cytokines that regulate the downstream immune responses and activate the lung immune network, which ultimately leading to BPD development. PAS results showed that glycogen content in lung tissue was reduced after ILC2 depletion and increased after ILC2 adoptive transfusion, which is consistent with the trend of Th17 cell response, indicating the pathobiology of the contribution of ILC2-Th17 cells in BPD. The above findings highlighted the imbalance of the ILC2-Th17 immune axis under hyperoxia exposure, and the critical role of IL-6 in orchestrating this cellular interaction.

This study has revealed that hyperoxia induces pulmonary ILC2 activation and a large amount of activated ILC2 participates in Th17 cell differentiation through the secretion of IL-6. ILC2-derived IL-6 regulated Th17 cell response and participated in the development of BPD. In addition, this study investigated the role of ILC2 depletion and adoptive transfusion in hyperoxia lung injury of BPD.

## Conclusions

In summary, our findings indicate that ILC2 regulates hyperoxia-induced lung injury via an enhanced Th17 cell response in the BPD mouse model (Fig. 9). The results suggest that the inhibition of the ILC2-Th17 immune axis can help to limit the development of BPD and may provide a new treatment strategy for BPD immunotherapies.

## Electronic supplementary material

Below is the link to the electronic supplementary material.

Supplementary Material 1

## Data Availability

The data presented in this study are available on request from the corresponding author.

## Abbreviations

BPD: bronchopulmonary dysplasia
ILC2: type 2 innate lymphoid cell
Th17 cell: T helper 17 cell
IL-17A: interleukin (IL)-17 A
IL-17F: interleukin (IL)-17 F
H&E staining: hematoxylin and eosin staining
RAC: radial alveolar count
RT-qPCR: Reverse transcription-quantitative PCR.
