# Supplementary material for: ILC2 regulates hyperoxia-induced lung injury via an enhanced Th17 cell response in the BPD mouse model
Source: BMC Pulm Med. 2023 May 30;23:188. doi: 10.1186/s12890-023-02474-9 (PMC10230686; doi:10.1186/s12890-023-02474-9)
Supplement: Supplementary file 1 — Supplementary Material 1 [file 12890_2023_2474_MOESM1_ESM.docx]

ILC2 regulates hyperoxia-induced lung injury via an enhanced Th17 cell response in the BPD mouse model

Yue Zhu^1^, Lanlan Mi^2†^, Hongyan Lu^1^* , Huimin Ju^1^, Xiaobo Hao^1^ and Suqing Xu^1^

^1^Department of Pediatrics, The Affiliated Hospital of Jiangsu University, No.438 Jiefang Road, Zhenjiang, Jiangsu 212001, China

^2^Department of Neonatology, Shanghai Children's Medical Center, No.1678 Dongfang Road, Pudong New Area, Shanghai 200127, China

^†^ These authors contributed equally to this work

* Correspondence: lhy5154@163.com; Tel.: +86-0511-8508-2260


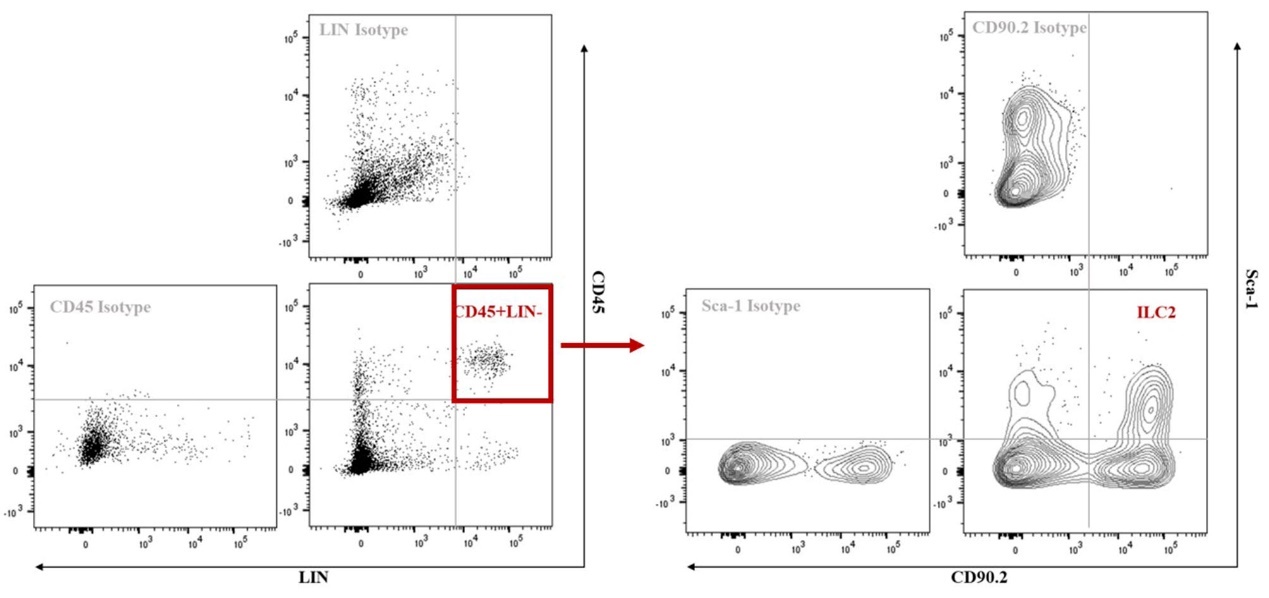
 **Supplementary Fig. 1** FMO controls in combination with isotype controls to check for non-specific binding of antibodies in the FACS. ILC2 is defined as CD45^+^Lin^-^CD90.2^+^Sca-1^+^.
